# Ehrlich’s “ 606 ”

**Published:** 1911-01

**Authors:** A. Samuels

**Affiliations:** Associate Professor of Gynecology, College Physicians and Surgeons, Baltimore, Md.


### Ehrlich's "606"

By A. SAMUELS, M.D.
Associate Professor of Gynecology, College Physicians and Surgeons, Baltimore, Md.

[*Maryland Medical Journal*, December, 1910]

THE discovery of "606" is the outcome of the labors of Dr. Ehrlich. For a long time he had been engaged in an effort to discover some chemical substance that would prove of value in the treatment of those infectious diseases caused by animal parasites, which apparently cannot be destroyed by the supplying of specific antibodies.

Dr. Ehrlich, from previous investigations, had found that it was not safe to attempt to destroy animal parasites by means of small doses of poisonous substances for the reason that the parasites often develop a toleration for the subtance which they transmit to their progeny. All of these chemical substances are more or less poisonous, attacking the protoplasm of the parasite as well as the organs of the body. Therefore, for a substance to be of therapeutic value in the treatment of those diseases caused by animal parasites, it must not be poisonous to the patient in doses required to kill the parasites.

With the discovery of the transmissibility of syphilis to the lower animals and of treponema pallidum, the parasitic cause of syphilis, these new drugs could be tested against the treponema pallidum in rabbits.

Through the aid of modern synthetical chemistry, he was able to prepare an almost unlimited number of combinations.

Among the many hundreds of synthetical combinations made and tested, it was found that the dioxydiamido arseno benzol would quickly kill all the spirochetæ in rabbits and was harmless to the host. To make it possible to introduce this remedy in the treatment of pathological conditions in man, Dr. Konrad Alt of Uchtspringe, made many experiments on paralytics and proved that the substance did not exert a poisonous effect in man in doses of from 0.3 to 0.5 gramme. Iverson, about this time, reported gratifying results in recurrent fever. Schreiber observed that the symptoms of human syphilis disappeared very quickly under treatment with the drug.

The substance "606" is chemically dioxydiamido arseno benzol; the active principle is arsenic and the other chemical groups present merely serve the purpose of fixing the base to the parasite. It is a yellowish powder which oxidizes on exposure to air, and forms a very poisonous substance. Hence, it is put up in vacuum tubes. It dissolves in water with difficulty, making a strongly acid solution. As the acid solution is very painful, it is best administered as a neutral base. Wechselman prepares the substance for injection by rubbing it in a mortar with 1 to 2 c.c. of sodium hydrate solution; then adding acetic acid drop by drop until a fine yellow paste is precipitated. This precipitate is made sterile and rubbed in 1 to 2 c.c. of sterile distilled water, and precisely neutralised to litmus with 0.1 normal sodium hydrate if acid, or a 1 per cent. acetic acid solution if alkaline. The precipitate in suspension is then drawn into a 5 c.c. syringe and injected subcutaneously either below the shoulder blades or in the gluteal region, the skin previously having been cleansed and disinfected.

*Effects of the Drug.*—The effects of the drug may be divided into those aising from the introduction of the compound into the system which are both local and constitutional and the effects of the drug on the pathological conditions caused by the animal parasites.

Results, locally. If the solution was precisely neutral, little or no discomfort was noted, severe pain only following in those cases when the solution was acid. In one patient, however, at the Virchow Hospital under the care of Dr. Wechselman, who suffered severely after the injection, it was necessary to administer morphine. A slight swelling generally arises on the second or third day following the injection. With the exception of one or two cases, the swelling subsided quickly. In one case, quite an induration appeared in the gluteal region which went on to abscess formation.

Constitutionally. A rise in temperature is generally noted. In some patients the temperature may reach 105°, especially in those who seem to have an arsenic idiosyncrasy. An urticarial eruption has also been noted.

In view of the experiences with other arsenical preparations, fear was entertained for the optic nerve, therefore, every patient has his eyes examined by a specialist before an injection is given. In cases in which there were changes in the optic nerve, injections were not given. Ehrlich claims from later experiments on animals that a toxic influence on the optic sphere is not very probable. Clinical observation at the Virchow Hospital seems to favor the opinion that "606" can be used even in cases of diseased optic nerve. For instance, in one patient whose eyes had not been examined, after the injection it was found that the optic nerve on each side had a dim margin and was venously hyperemic (neuritis optical). On investigation it was found that she had been treated by inunctions of mercury and had at that time a dim margin. The patient remained without any pathological affection of the fundus oculi and retained her full vision (Dr. Fehr).

Of the number of patients that I saw treated at the Virchow Hospital, ocular affections were not observed and Wechselman expressed himself by saying he did not believe it injudicious to use "606" when the retina is affected. However, he always has the eyes, heart and kidneys examined by a specialist before giving them an injection of "606."

The Effects of "606" in Syphilis. So far, Wechselman has treated over 700 patients, and during my stay at the Hospital, I saw about 40 patients treated. The results obtained from the injections in these 40 patients correspond in general to the results obtained from the sum total treated by Wechselman. From 12 to 24 hours after an injection, erosive chancres become clean and rapidly heal; in pronounced sclerosis, the cleaning process is of the same rapidity, but absorption takes longer. I understand that in four cases there appeared, after healing of the primary lesion, an exanthem which disappeared spontaneously in two patients in whom it appeared on the first and seventh day after the injection; the other two patients received a second injection. Mucous patches of the mouth heal in from 24 to 48 hours, even in patients who are inveterate smokers. The roseola disappeared in a few days. Malign ulcerous syphilides, rupia, watery pustules and small papulous syphilides are slower in their disappearance and a second injection sometimes becomes necessary. The night pains so common with syphilis of the bones disappear with great rapidity; Wechselman explains the sudden stopping of the pains to the killing of the spirochetæ. Visceral lues show quick recovery, especially syphilis of the testicles. A case of long standing icterus disappeared in about ten days. Syphilitic growth of the larynx which had produced a severe dyspnea and stridor disappeared quickly after an injection and remained only as a solid infiltration and was treated with a second injection. Edema of the larynx did not occur. Severe constitutional symptoms were noted in a case of cerebral lues; temperature 106, pulse 140, with extreme prostration that lasted five days. The cerebral lues had not shown a decided improvement. A remarkable effect was observed in one patient suffering from cerebral lues. For six months this patient had been under mercurial injections, the lues contracted eight years previously. He had double vision with paresis of the abducen with diminution of vision of the right eye, the left being perfect. He was given 0.5 grammes of "606"; until the seventh day improvement was not apparent. After the seventh day of injection, a remarkable improvement seemed to have taken place over night, as he could clearly recognise the newspaper print and the contour of buildings with an accelerated pupillary and ciliary action to light.

Parasyphilitic diseases (tabes, paresis, and the like) so far have shown little or no improvement. Some symptoms seemed to improve, such as the girdle sensation in tabetics, but this probably would have occurred in this class of patients with most any injection. The Wasserman reaction usually disappears in from eight to forty days. In one or two cases of absolute lues with a negative Wasserman reaction, a positive reaction appeared several days after the injection and then disappeared.

Wechselman explains this obscure reaction to the setting free of quiet spirochetæ by the injection and they carrying their specific matter into the circulation. Widespread and severe recurrences, such as occur after mercurial treatments, have not been noted. In a few cases of recurring syphilis, a second injection has been given.

